# Supplementary material for: Patterns of Intron Gain and Loss in Fungi
Source: PLoS Biol. 2004 Nov 30;2(12):e422. doi: 10.1371/journal.pbio.0020422 (PMC532390; doi:10.1371/journal.pbio.0020422)
Supplement: Table S1 — Also available at http://genes.mit.edu/NielsenEtAl/. (4.3 MB ZIP). [file pbio.0020422.st001.zip › NielsenEtAl/html/1354.html]

AN5206.1.NCU02954.1.MG01566.1.FG01392.1


```
 CLUSTAL W (1.82) Multiple Sequence Alignments - Introns Inserted


Sequence 1: NCU02954.1	357 aa
Sequence 2: FG01392.1	356 aa
Sequence 3: MG01566.1	360 aa
Sequence 4: AN5206.1	360 aa
Alignment Length: 363 aa
Number Identitical Residues: 254 aa
Alignment Score (without introns) 10387


MG01566.1 	MATARALRIG~L2IAGDGIGKDVVP~AGRRVLEALPASLGLRFSFVDLQAGFQTFEETGS
NCU02954.1	-MAARTLKIG1L~IPGDGIGKEVIP~AGRRILEALPASLGLKFEFIDLKAGWETFEQTGN
FG01392.1 	-MSFRTLRIG1L~IPGDGIGKEVIP~AGRRILEALPSSLGLKFDFVNLKAGFETFEQTGA
AN5206.1  	MAAARTLRIG1L~IPGDGIGREVIP0AGRRILEALPASLNLKFNFVDLDAGYDCFKRTGT
          	  : *:*:** * *.*****::*:* ****:*****:**.*:*.*::*.**:: *:.** 

MG01566.1 	ALPDRTVETLKGECDGALFGAVS~SPTKPVKGYSSPIVGLRKKLDLYANVRPVKTVVS--
NCU02954.1	ALPEETVSILKSECDGALFGAVS~SPTKAVKGYSSPIVALRKKLDLYANVRPVKSVRGP-
FG01392.1 	ALPDKTVEVLKNECDGALFGAVS~SPTQAVKGYSSPIVALRKRLDLYANVRPVKTVMT--
AN5206.1  	ALPDKTVEVLKKECDGALFGAVS2SPSTKVAGYSSPIVALRKKLDLFANVRPVKTTAGTS
          	***:.**. ** *********** **:  * *******.***:***:*******:.  .:

MG01566.1 	APRPIDMVIVRENTEDLYVKDERTFDAPDGSGPVAEAIKRISYRASHRIASMAGEIALRR
NCU02954.1	GVKPIDMVIVRENTEDLYVKEEKTYDTPEGK--VAEAIKRISQRASSRIATIAGEIALRR
FG01392.1 	AAKPIDMVIVRENTEDLYVKQEKTYDTPEGK--VAEAIKRISEKASFRIAAMAGDIALRR
AN5206.1  	AGKPIDLVIVRENTEDLYVKEESTEETPNGK--VARAIKQISERASSRIATIAGEIALRR
          	. :***:*************:* * ::*:*.  **.***:** :** ***::**:*****

MG01566.1 	QKIRAAGAPS-IHSSPLVTITHKSNVLSQTDGLFRSTAKEALADPKYAGSVAVEEQIVDS
NCU02954.1	QKIREGGSPS-IHKGPLVTVTHKSNVLSQTDGLFRSTAREALAAGRFT-SVAVEEQIVDS
FG01392.1 	QKIRDAGASS-IHKSPLVTITHKSNVLSQTDGLFRATSKRALADPKFS-SVAVEEQIVDS
AN5206.1  	QNIRDGAAASGLRTKPMVTITHKSNVLSQTDGLFRETARAALAAQKFS-SVEVEEQIVDS
          	*:** ..:.*.::. *:**:*************** *:: ***  ::: ** ********

MG01566.1 	MVYKLFRQPEDYDVIVAPNLYGDILSDGAAALVGSLGLVPSANVGDGFVMGEPCHGSAPD
NCU02954.1	MVYKLFRQPEAYDVIVAPNLYGDILSDGAAALVGSLGLVPSANVGEGFAIGEPCHGSAPD
FG01392.1 	MVYKLFRQPEDYDVIVAPNLYGDILSDGAAALVGSLGLVPSANVGEGFAIGEPCHGSAPD
AN5206.1  	MVYKLFRQPEYYDVIVAPNLYGDILSDGAAALVGSLGLVPSANVGDNFAIGEPCHGSAPD
          	********** **********************************:.*.:**********

MG01566.1 	IMGKNIANPIATLRSAALMLEFLNEEEAAAKIYAAVDANLVEGKVLSPDLGGKATTDQVL
NCU02954.1	IQGQNIANPIATLRSAALMLEFLNEEEAAAKIYAAVDANLEEGKLLSPDLGGKAKTEEVV
FG01392.1 	IQGQNIANPIATLRSTALMLEFLNEEEAAAKIYAAVDGNLEDGKLLSPDLGGKATTEEVV
AN5206.1  	IEGKNIANPIATLRSVALMLEFLGEEQAAAKIYAAVDGNLDEGKYLSPDMGGKATTTEVL
          	* *:***********.*******.**:**********.** :** ****:****.* :*:

MG01566.1 	EDILRRL
NCU02954.1	QDILRRL
FG01392.1 	ADILRRL
AN5206.1  	EDVLKRL
          	 *:*:**
```
